# Endoscopic transaqueductal stent placement for tumor-related aqueductal compression in pediatric patients: surgical consideration, technique, and results

**DOI:** 10.1007/s00381-023-06171-0

**Published:** 2023-10-12

**Authors:** Anna Prajsnar-Borak, Henry W. S. Schroeder, Joachim Oertel

**Affiliations:** 1 Department of Neurosurgery, Saarland University Medical Center, and, Faculty of Medicine, University of Saarland, Kirrbergerstraße, Building 90.5, D-66421 Homburg, Germany; 2 Department of Neurosurgery, Ernst Moritz Arndt University, Greifswald, Germany

**Keywords:** Pediatric, Aqueductal stenosis, Intraventricular tumor, Neuroendoscopy

## Abstract

**Purpose:**

Endoscopic transaqueductal stenting has become a well-accepted treatment option for a selected small subset of aqueductal stenosis-related obstructive hydrocephalus. However, transaqueductal stenting poses unique challenges and risks which requires critical consideration. This report discusses the clinical experiences with transaqueductal stenting for periaqueductal tumor-related aqueductal stenosis focusing on pediatric patients.

**Methods:**

A retrospective analysis of all patients undergoing endoscopic TAS from 01/1993 to 01/2022 in the author’s departments was performed. Demographic, clinical, radiological, and intraoperative endoscopic data were evaluated. All patients with AS-related occlusive hydrocephalus that was treated with TAS were analyzed and prospectively followed. Special attention has been given to providing insights into indications, surgical technique, and limitations.

**Results:**

Out of 28 endoscopic transaqueductal endoscopis stenting procedures, five procedures were performed on periaqueductal tumor-related obstructive hydrocephalus, two children and three adult patients. CSF pathway was obstructed by tumor located in the aqueduct in 2, by tumor in the thalamus/mesencephalon in 1, by a tumor within the third ventricle in 1, and by a tumor of the lamina tecti in 1. Simultaneously with transaqueductal stenting, 2 endoscopic third ventriculostomies (ETV), 3 tumor biopsies, and 1 tumor resection were performed. Postoperative complications included the following: CSF fistula (1 case), and asymptomatic fornix contusion (1 case). A working aqueductal stent was achieved in all cases based on clinical follow-up evaluation. Postoperatively, all patients showed improvement or resolution of their symptoms. The mean follow-up period was 25.2 months (range, 1–108 months). One patient died due to tumor progression during early follow-up. No stent migration was seen.

**Conclusion:**

Endoscopic third ventriculostomy remains the gold standard for treatment of CSF circulation obstructions with lesions in the posterior third ventricle and aqueduct. Transaqueductal stenting for periaqueductal tumor-related aqueductal compression is technically feasible. However, because of the potential high risks and subtle advantages compared with ETV transaqueductal stenting, it might be indicated in a small subset of well-selected patients if alternative treatment options are not at hand.

## Introduction

Successful treatment of aqueduct stenosis (AS)-related non-communicating hydrocephalus due to its peculiar characteristics and variable etiology can be highly challenging. Various endoscopic techniques and treatment options for CSF restoration in AS have been reported [1–19]. Three main endoscopic techniques, namely aqueductoplasty, transaqueductal stenting (TAS), and fenestration into the lateral ventricle, have been proposed [20–23]. Endoscopic TAS has become a valuable treatment option for a selected subset of AS [2, 3, 20, 24, 25] and favorable clinical and radiological results have been recently reported [1, 5, 14, 20, 25–29]. The outcome and prognosis of patients with aqueductal stenosis are highly variable and depending on several factors. The success rate is highly influenced by the mechanism of obstruction, the morphology of aqueductal obstruction, and the patient’s age [9, 14, 17, 24, 30–32]. Nevertheless, endoscopic TAS represents a therapeutic option, which could be considered in each case of compatible intraventricular and aqueductal anatomy [20] or secondary after failed ETV by periaqueductal tumors-related aqueductal compression [3]. On the contrary, most authors recommend ETV for membranous or tumor-related aqueduct as the first-line treatment before shunting or stenting procedure for aqueduct-related CSF impairment [5, 7, 10, 33]. Uniform consensus regarding the best endoscopic approach fails, and the debate concerning the optimal technique paradigm for periaqueductal tumor-related AS-related CSF impairment remains unsolved. Data on endoscopic TAS by tumor-related AS in pediatric patients is still limited to relatively small case series (Table 1). Surgical consideration for intraventricular periaqueductal tumor-related aqueductal compression in pediatric patients would be desirable. The authors present their surgical experience with endoscopic TAS in tumor-related aqueductal stenosis, focusing on indications, surgical technique, technical nuances, limitations, and complications.

**Table 1:** Literature review regarding the endoscopic transaqueductal stenting for periaqueductal tumor-related aqueductal stenosis

**Series**	**Number of patients**	**Patients group**	**Indications for TAS**	**Endoscope used**	**Approach**	**Results**	**Approach-related complications**	**Follow-up**
Bulsara et al. [3], 2003	1	Children	Tectal tumor (1) bei Failed ETV	NA	Frontal burr hole	Aqueductal stenting may be alternative to ETV or shunts in some patients with tectal tumor-related AS	None	12 months
Fritsch et al. [34], 2004	27	Children	Membranous distal AS (5) Periaqueductal tumor (4) IFV ( 18)	Rigid lens scope (Aesculap, Mionop, or Paediscope) A flexible endoscope (Storz)	Frontal precoronal burr hole	In patients with tumor-associated AS, aqueductoplasty alone will not stay open. Those patients would better be treated with ETV Patients with IFV have a significant restenosis rate after aqueductoplasty. Therefore, aqueduct stenting is recommended	Two transient and one permanent oculomotor nerve palsy One asymptomatic posterior fossa subdural hygroma	24 months
Geng et al. [26], 2015	8	Adults and children	Intraventricular tumor (3) Intraventricular cysticercosis (2) Membranous AS (3)	6 mm diameter rigid lens (LOTTA, Karl Storz, Tuttlingen, Germany)	Frontal bur hole	All patients showed improvement, no recurrence of aqueductal obstruction	Stent migration (1 case) Transient mutism (2 cases) Transient oculomotor nerve palsy (1 case)	27 months (1–51 months)
Our series	5	Children (2) Adults ( 3)	Periaqueductal tumor (5)	Gaab and Flexible endoscope, (Karl Storz, Tuttlingen, Germany	Frontal precoronal	Optimal TAS was achieved in all cases. Simultaneously with TAS, 2 ETV, 3 tumor biopsies, 1 tumor resection, and 1 aqueductoplasty have been proceeded. Postoperatively, all patients showed improvement or resolution of their symptoms, 1 patient died due to tumor progression	Asymptomatic fornix contusion ( 1 case) CSF fistula (1)	25,2 months (1–108 months)

*NA* not available, *AS* aqueductal stenting, *VC* ventricle catheter, *IFV* isolated fourth ventricle, *ETV* endoscopic third ventriculostomy

## Methods

### General

Out of 28 endoscopic TAS procedures, 5 (2 pediatric, 3 adults) surgeries were considered for CSF impairment due to periaqueductal tumors-related aqueductal compression, and performed between March 1993 and February 2003 at the Department of Neurosurgery of the Ernst Moritz Arndt University, Greifswald, Germany, and between December 2010 and March 2023 at the Department of Neurosurgery of the University of Saarland, Homburg, Germany. Clinical and radiologic follow-up evaluation was documented in serial examinations. The clinical patients’ data and radiologic characteristics, focusing on assessing the configuration of the aqueduct and foramen of Monro anatomy, and tumor location, are given in Table 2. Each evaluated case was prospectively followed by clinical-neurological and radiologic examinations (magnetic resonance (MR) imaging or computed tomography). The Gaab Universal Neuroendoscopic System, or flexible endoscope (Karl Storz GmbH & Co.KG, Tuttlingen, Germany), was used. The intraoperative data have been acquired from the department’s internal database. The positioning of the transaqueductal catheter was assessed using postoperative radiological studies (MR images or computed tomography). The early functionality of aqueductal stenting was assessed based on a comparison of preoperative and postoperative ventricle size in achieved MR images or computed tomography.

**Table 2:** Descriptive summary of patients’ data

**Patient No**	**Age/se**x	**Main clinical symptoms**	**Tumor location**	**Main endoscopic procedure/endoscope used**	**Concomitant procedure**	**Histology**	**Navigation**	**Side of the approach**	**Foramen Monro**	**Complications related to endosc. technique**	**Postop. clinical symptoms**	**Radiological improvement, a decrease in ventricle size**	**Shunt-dependent**	**Outcome, at last, follow-up** **(months)**
1	41/M	Visual impairment	Aqueduct	TAS (Gaab, flexible endoscope, 0-, 30, 70-degree optik)	ETV Tumor resection Aqueductoplasty	Gliosis	Used	Right	Sufficient 6–10 mm	Asymptomatic fornix contusion	Asymptomatic	Evident	No	2/asymptomatic
2	8/M	Headache, nausea vomiting	Aqueduct	TAS Gaab I, 0-, 30-, 70-degree optik	Tumor biopsy	Gliosis	No	Left	Wide, Above 10 mm	No	asymptomatic	Evident	No	108/asymptomatic
3	35/M	Double vision due to CN 6 palsy	Thalamus/mesencephalon	TAS Gaab I, 0-, 30-, 70-degree optik	Tumor biopsy	Astrocytoma, WHO grade II	Used	Left	Sufficient 6–10 mm	No	Asymptomatic	Evident	No	1/asymptomatic
4	3/M	Visual impairment, headache, nausea, vomiting	Third ventricle	TAS Gaab II, 0-degree optik	-	Neuroblastoma	No	Left	Sufficient 6–10 mm	CSF fistula	Idem	Idem	Yes	1/asymptomatic Died due to tumor progress
5	25/F	Double vision due to CN 6 palsy	Lamina tecti	TAS Gaab I, 0-, 30-, 70-degree optik	ETV Tumor biopsy	Astrocytoma, WHO grade II	No	Left	Wide 10 mm	No	Asymptomatic	Evident	No	14/asymptomatic

*M* male, *F* female, *y* years, *TAS* transaqueductal stenting, *ETV* endoscopic third ventriculostomy, *AP* aqueductoplasty, *CN* cranial nerve

### Surgical technique and equipment

All procedures were performed under general anesthesia. The patients were positioned supine with the head slightly retroflected and fixed in a Mayfield headrest system. Preoperative evaluation of MR images was of utmost importance in assessing the configuration of foramen Monro’s, aqueduct’s entrance anatomy, intraventricular tumor location its size, and site, influencing the considering of the endoscopic technique choice. The decision to perform ETV, tumor biopsy, and TAS through a single burr hole or through two separate burr holes was predominantly dependent on tumor location, its size, width of the foramen of Monro, distortion of the aqueduct, and the degree of ventricle enlargement. Whenever possible, a right-sided approach was used. However, if the right foramen of Monro was narrowed or distorted, or the periaqueductal tumor was localized mostly right-sided, a left-sided approach was chosen. The approach via a trajectory from skin incision through a lateral ventricle, foramen of Monro, the floor of the third ventricle, and an aqueduct was critically calculated.

If necessary, neuronavigation for the planning of entry point, and trajectory for aqueductal stenting, tumor biopsy, or tumor resection, was considered. Neuronavigation has been particularly helpful in patients with small ventricles. In most cases, the burr hole was placed between 3.0 and 5.0 cm anterior to the coronal suture and about 2.0 cm lateral from the midline. In cases where a concomitant endoscopic procedure, such as ETV, tumor biopsy, or any other endoscopic procedure was considered, the initial approach was adjusted accordingly, or a separate burr hole was created, if necessary. The endoscope was inserted into the ventricle through a trajectory offering the optimal working space. For the endoscopic setting, Gaab Universal Neuroendoscopic System, a flexible endoscope (Karl Storz GmbH&Co., Tuttlingen, Germany) was considered. A detailed description of the instruments and the general endoscopic technique has been published previously [35]. Succinctly, once the dura was incised, the operating sheath with the trocar was introduced freehand into the lateral ventricle and fixed with two retractor arms after achieving the optimal position. The trocar was withdrawn, and the rigid rod-lens diagnostic scope was inserted. The lateral ventricle was explored. The operating sheath was applied to the third ventricle. The third ventricle’s floor and the aqueduct’s entrance were explored. In most presented cases, this technique was feasible because the foramina of Monro were sufficiently dilated. Finally, the diagnostic scope was replaced when the operating scope was introduced. If indicated, the concomitant ETV was performed. The perforation of the floor was made just behind the clivus, halfway between the infundibular recess and mammillary bodies in the midline. The blunt perforation was assessed using standard instruments such as balloon catheter and Decq forceps allowing precise and atraumatic perforation. Thereafter, if considered, tumor biopsy proceeds. The endoscope was positioned in front of the lesion. In the presented series, the tumors were located in the periaqueductal space, the ventricle, or lamina tecti, so by sufficient size of Foramen of Monro, all considered procedures were performed via a one burr hole which was located approximately 3.0 to 5.0 cm in front of the coronal suture. A fine and gentle tilt enabled access to both targets: the floor of the third ventricle and the tumor. Finally, after completing the tumor biopsy, the endoscope can be positioned caudally for performing TAS. If necessary, the configuration of the aqueduct and the fourth ventricle were additionally inspected using a 2.5-mm steerable, flexible scope to evaluate aqueductal patency and, if existing intraaqueductal membranes, mechanical perforation using a flexible endoscope was performed. The preoperative prepared aqueductal stent with multiple proximal and distal-created side holes was stepwise inserted into the fourth ventricle through the working channel of the endoscope. The final catheter positioning was adapted under endoscopic guidance. The length of the stent was preoperatively calculated based on the preoperative MR images. The stent was fixed to a burr hole reservoir to avoid a possible stent migration. Combine technique with the additional usage of a flexible endoscope concomitantly to Gaab scope was considered in difficult distorted anatomical circumstances, using simultaneous beneficial aspects of both endoscopes and counterbalancing their limitations.

## Results

Among the 5 studied patients, two of them were children: two male patients (mean age 5,5 years; range 3–8 years) and three adult patients: two male and one female patient (mean age 33.6 years; range from 25 to 41 years). All patients suffered from signs of elevated intracranial pressure due to CSF impairment. The tumor was located in the aqueduct (2 cases), in the thalamus/mesencephalon (1 case), in the third ventricle (1 case), and in the lamina tecti (1 case). The underlying pathology was astrocytoma, WHO, grade II, (2 cases), gliosis (2), and neuroblastoma (1 case). The main clinical presentation was signs of increased intracranial pressure symptoms with headache (40%, 2 of 5 cases), nausea, vomiting (40%, 2 of 5 cases), double vision due to abducens nerve palsy (40%, 2 of 5 cases), and visual impairment (1 case, 20%). All patients underwent a preoperative MR image examination. All patients had significantly enlarged lateral and third ventricles due to AS at the time of MR image acquisition. The details of all patients are summarized in Table 2. All studied patients were considered for endoscopic transaqueductal stenting. A successful endoscopic TAS was achieved in all 5 procedures. In four surgeries, additional concomitant endoscopic procedures were considered: 3 tumor biopsies, 2 ETVs, 1 tumor resection, and 1 enlargement of aqueduct by balloon catheter (aqueductoplasty). The neuronavigation was used in two procedures. Because of intraoperative anatomy and periaqueductal tumor location, the left-sided pre-coronal approach was used in 4 procedures and the right-sided in 1 surgery.

Concerning the intraventricular anatomy, and planned simultaneous procedure, in four surgeries, a standard Gaab neuroendoscope with 0-, 30-, and 70-degree scope was used. In one case, Gaab scope and flexible endoscope were concomitantly used. The Gaab rigid rod-lens scope was initially used for the inspection and exploration of the third ventricle and the aqueductal entrance. The 2.5-mm steerable, flexible endoscope was additionally used to widen the gliotic narrowing and to explore the fourth ventricle before stent placement. Additional insertion of angled telescopes (30°, 45°, 70°), rotated through 360°, enabled us to gain a wide intraventricular panoramic view, to explore and intraoperatively assess the distorted by tumor intraventricular anatomy and the morphology of aqueductal entrance, prior to tumor biopsy and aqueductal stenting. The stent was stepwise protruded transaqueductal into the fourth ventricle through the working space of the operating Gaab endoscope in a “two-in-one technique” to get control over the transaqueductal stent insertion. The aqueductal stent was stepwise inserted into the fourth ventricle through the working channel.

### Configuration of foramen of Monro and the entrance of the aqueduct

Considering the morphological circumstances, a large foramen of Monro (above 10 mm) was seen in 2 cases, and acceptable sufficient size (6–10 mm) in 3 cases in the presented series. The aqueductal entrance was highly compressed by the tumor in all 5 cases.

### Patients and follow-up

The mean follow-up period was 25.2 months (from 1 to 108 months). In the initial postoperative course, all studied patients improved relevant clinically (5 patients, 100%), and 4 of 5 patients (80%) showed radiological improvement with a sufficient decrease in ventricle size. There was no procedure-related mortality and no permanent morbidity. The number of total complications was 32.14% (2 of 5 procedures), with 1 complication being asymptomatic (1 case of asymptomatic fornix contusion and 1 case of CSF fistula). One patient was shunt-dependent and after 1 month died due to tumor progression. No stent migration or dislocation was observed. After an average follow-up, the functionality of the transaqueductal stent was obvious in all patients.

### Exemplary case

**Fig. 1:**
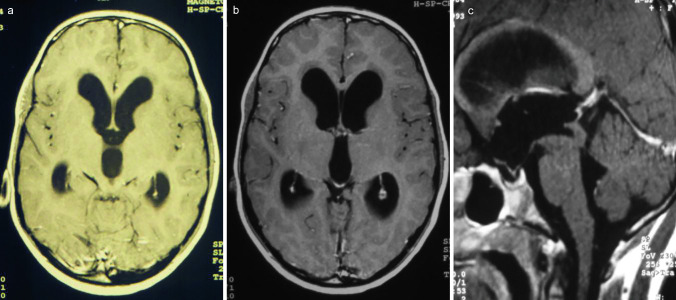
Preoperative axial **a**, **b**, and sagittal **c** MR images showing tri-ventricular occlusive hydrocephalus due to compression of the aqueduct by a tectal lesion, suspected for low-grade tumor

**Fig. 2:**
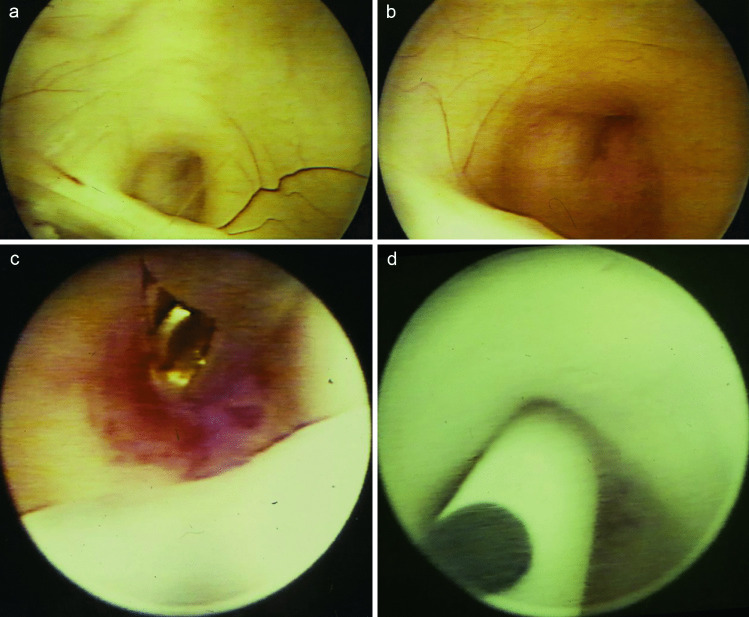
Intraoperative endoscopic view on the occluded aqueduct above the posterior commissure **a**, **b**. After an endoscopic biopsy **c**, the transaqueductal stent is inserted via the aqueduct into the fourth ventricle **d**

**Fig. 3:**
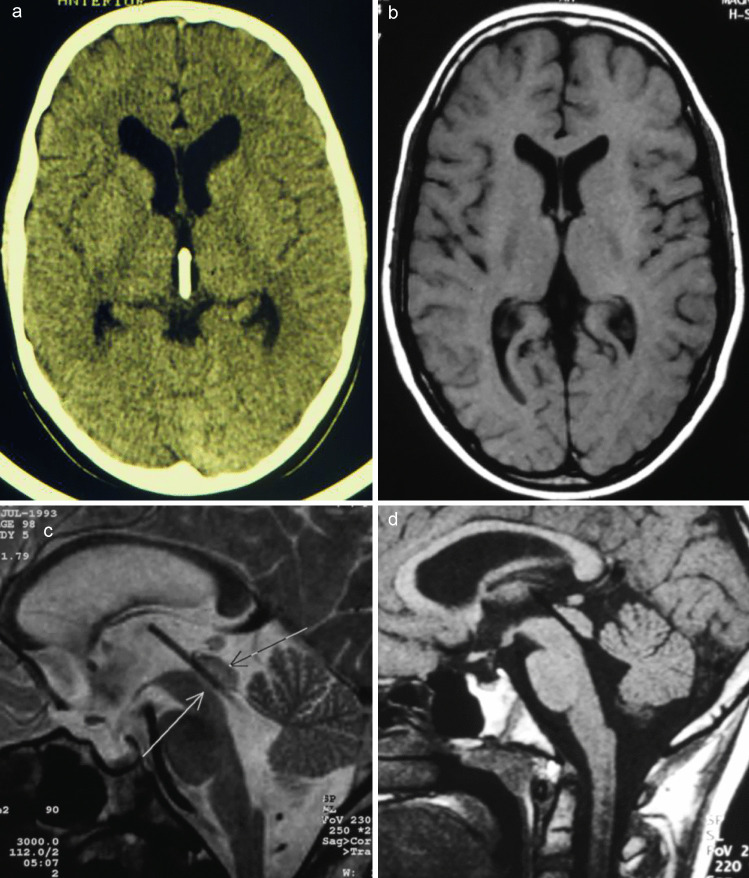
Postoperative obtained axial computed tomogram **a** axial and sagittal MR images **b**, **c**, **d** showing the optimal positioning of the transaqueductal stents. Additionally, the obvious reduction of the lateral and the third ventricle size indicated the functionality of the stent

**Fig. 4:**
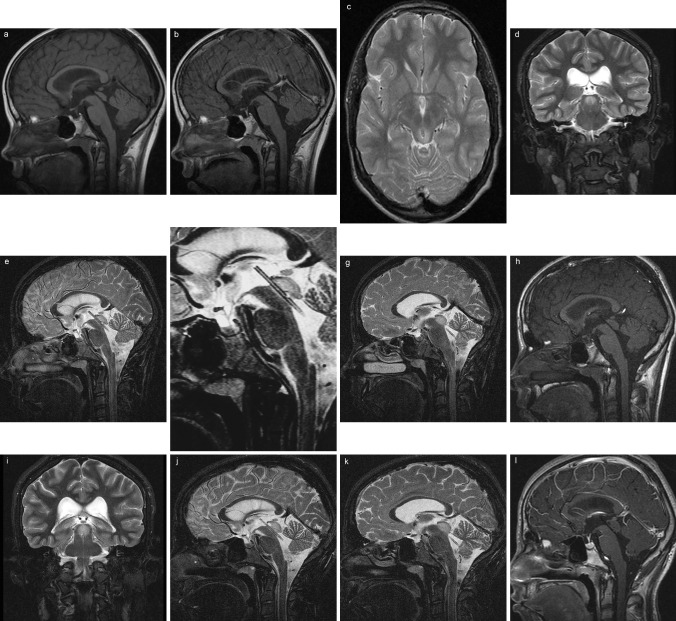
Postoperative MR images obtained 2 years **a**, **b**, 6 years **c**, **d**, **e**, **f**, **g**, **h**, and 9 years **i**, **j**, **k**, **l** after transaqueductal stenting, respectively, showing consistently the optimal position of the stent and its functionality

An 8-year-old boy, suffering from progressing signs of elevated CSF pressure with headache, vomiting, and nausea, was admitted emergently at our department. The initial MR images showed a triventricular obstructive hydrocephalus with a periaqueductal tumor compressed to the lumen of the aqueduct (Fig. 1). The decision of endoscopic ETV, tumor biopsy, and trans-aqueductal stent placement was made (Fig. 2). Postoperative computed tomogram and MR images were obtained 1 day after surgery (Fig. 3) confirming the optimal position and functionality of the transaqueductal stent. The size of the lateral and the third ventricle regressed revenant. Directly after surgery, the CSF elevation-related symptoms resolved completely. Until the last follow-up, no catheter revision was required. During the last follow-up of 108 months, a symptom-free clinical course and a satisfied radiological course were objectivized (Fig. 4).

## Discussion

Endoscopic technique for CSF restoration is the procedure of choice for many aetiologies of AS-related non-communicating hydrocephalus. Although ETV is well established for many forms of tumor-related aqueductal stenosis and is considered a safe and effective procedure [2, 4–6, 8, 11–13, 19], some potential critical complications are known [36, 37]. Anatomical variations and distortion of the third ventricle floor and the aqueductal entrance due to tumors, membranes, or inflammatory sequelae may cause the failure of ETV. On the other hand, although aqueductal stenting is an alternative treatment option for ETV in non-neoplastic and neoplastic AS, most authors prefer an ETV, which eliminates the need for using any foreign body material and decrease the risk of infection or malfunction [5, 10, 42]. Endoscopic aqueductal stenting has become a valuable alternative for a selected subset of AS. AS by isolated fourth ventricle in children and young adults is one of the most commonly accepted indication for endoscopic TAS [5, 14, 20, 25, 27–29, 34, 38–40]. The best treatment option for other forms of CSF impairment at the level of the aqueduct, such as periaqueductal tumors-related aqueductal compression in pediatric patients, remains controversial. Most authors recommend ETV for tumor-related or membranous AS as the first-line treatment before shunting or stenting [5, 7, 10, 33, 39]. Uniform consensus regarding the best endoscopic approach fails, and the debate concerning the optimal technique paradigm for periaqueductal tumor-related AS-related CSF impairment remains unsolved (Table 1). Geng and colleagues [26] reported on 8 patients (children and adults) who underwent endoscopic TAS. In three of these cases, the aqueductal stent was indicated by intraventricular tumor-related AS and was successfully placed using a rigid lens Lotta endoscope through a frontal burr hole approach. After an average follow-up of 27 months, a recurrence of aqueductal obstruction has not been observed. Fritsch et al. [34] evaluated whether the long-term interventricular communication following aqueductoplasty is determined by the etiology of the AS. Out of four children with AS due to a periaqueductal tumor, three of them underwent aqueductoplasty and tumor biopsy with subsequent observed restenosis of the aqueduct. One child underwent aqueductoplasty with a stent and tumor biopsy at the same time. The authors postulated that in patients with tumor-associated AS aqueductoplasty alone will not stay open and those patients would better be treated with third ventriculostomy. Bulsara et al. [3] reported on a 12-year-old child presented with obstructive hydrocephalus due to aqueductal compression by tectal tumor. Five months after initially performed ETV came to recurrence of symptoms by failed ETV. The indication for aqueductoplasty and aqueductal stenting was considered. In the 1-year follow-up, an asymptomatic clinical course and the functionality of the stent in the final MR images were documented. The summary of endoscopic TAS procedures in children is summarized in Table 1. In the current paper, we discuss our experience gained with endoscopic TAS in 2 children and 3 adult patients. CSF impairment at the level of aqueduct was caused by a tumor located in the aqueduct (2), thalamus/mesencephalon (1), and third ventricle (1) lamina tecti (1). In pediatric patients, intraventricular tumors are often a cause of obstructive hydrocephalus. Therefore, the two main therapeutic strategies, namely the re-establishment of CSF circulation and gaining the histopathological tissue for further therapy, are crucial in surgical decision-making. The location of the tumor, the site of attachment of the tumor to the surrounding intraventricular structures, and the degree of aqueductal compression should be carefully analyzed. Considered approach and trajectory for tumor biopsy and aqueductal stent placement is determined by a few factors mentioned above.

Patient selection is a crucial aspect of successful endoscopic TAS procedures and surgical outcomes with low complication rates. In our opinion, the tumors located in the distal part of the third ventricle and periaqueductal region can be successfully reached from the contralateral transventricular approach, enabling the direct view of the tumor. Through the same corridor, the aqueductal stenting can be placed. Profound anatomical orientation plays an even more significant role. In the distorted by tumor intraventricular anatomy, narrowing the distal part of the third ventricle and aqueductal entrance, additional use of a flexible endoscope may be very beneficial because of intraluminal application and lesser invasiveness in comparison to a standard rigid endoscope. All these conditions may lead to incorrect aqueductal stenting placement or may pose some danger due to injury of the important intraventricular structures and cerebral aqueduct. Using Gaab endoscope with angled telescopes (30°, 45°, 70°), for direct visualization of the tumor and aqueductal entrance and rotated through 360°, enables to gain a wide intraventricular panoramic view, and maximized diagnostic efficacy. The use of concomitant Gaab, rigid scope, and flexible endoscope for intraventricular exploration are effective and help to overcome the shortcomings and some limitations of each endoscope used solely. The value of neuronavigation for aqueductal stenting in some particular indications should be separately stressed. The combined use of frameless neuronavigation for neuroendoscopy and occlusive hydrocephalus has been reported previously [41]. In our series, neuronavigation was additionally applied in two procedures. Neuronavigation was of value in calculating the ideal trajectory for the aqueductal stenting combined with other endoscopic procedures such as ETV, tumor biopsy, or tumor resection even in far distal locations such as lamina tecti or aqueduct. Concomitant performing of ETV, tumor biopsy, and TAS requires careful preoperative image evaluation. A decision to perform these endoscopic procedures through a single bur hole or two separate bur holes is dictated by the tumor location in relation to massa intermedia and tumor size. In these cases, the exact location of the burr hole is determined by the intersection of ETV, tumor biopsy and ETV, and aqueductal stenting, using neuronavigational guidance. Another aspect to consider in regard to planning the entry side is the laterality of the tumor. A contralateral, cross-over approach for eccentric intraventricular tumors may allow in some cases for the most direct trajectory. It is also important to stress that blockade of the aqueduct by a tumor or its distortion is a serious condition and endoscopic stenting should be only performed when the anatomic landmarks are evidently identified. In case, when there is any doubt, ventriculoperitoneal shunting or ETV should be considered.

Apart from the variability of the aqueductal entrance, the aqueduct has often a curved shape. Placing the burr hole approximately 5 cm anteriorly to the coronal suture and planning the straight-line trajectory through the foramen of Monro to the aqueduct’s entrance may pose a reliable danger of tectal plate roof injury. In these circumstances, aqueductoplasty for slightly inflating the aqueductal entrance may be an option before stenting.

The concomitant application of a flexible endoscope offers some additional advantages. The main reason is the superiority in anatomical exploration of the aqueduct and the fourth ventricle. Additionally, the flexible endoscope was used in one procedure to perforate the intraaqueductal gliotic membranes before the stenting. The stent was inserted into the aqueduct via the main working channel of the operating endoscope. The flexible endoscope was implemented through the working space of the operating endoscope. Using the combined endoscopic technique for intraventricular exploration, orientation, and assistance reason was effective and feasible. It helped to optimize the surgical effectiveness using each of the endoscope’s inherent advantages while counterbalancing and overcoming the limitations of each endoscopic visualization technique. Apart from the technique used, intraoperative complications are not always avoidable in some difficult circumstances mentioned above. In our series, we faced mild fornix contusion without postoperative clinical manifestation (1 case). The successful transaqueductal stent placement was achieved in all presented cases. No technique-related morbidity was observed.

## Conclusions

Endoscopic transaqueductal stent placement in patients with tumor-related aqueductal stenosis is a feasible treatment option in a well-selected small group of patients. Its risks are acceptable in experienced hands. However, ETV remains the gold standard for the treatment of obstructive hydrocephalus due to third ventricle tumors. It should be preferred whenever possible.

## Data Availability

The original data of this publication are available upon request.

## Abbreviations

TAS: Transaqueductal stenting
CSF: Cerebrospinal fluid
ETV: Endoscopic third ventriculostomy
AS: Aqueductal stenosis
MR: Magnetic resonance
